# A qualitative interview study of Australian physicians on defensive practice and low value care: “it’s easier to talk about our fear of lawyers than to talk about our fear of looking bad in front of each other”

**DOI:** 10.1186/s12910-022-00755-2

**Published:** 2022-03-04

**Authors:** Nola M. Ries, Briony Johnston, Jesse Jansen

**Affiliations:** 1 Faculty of Law, University of Technology Sydney, PO Box 123, Sydney, NSW 2007 Australia; 2 School for Public Health and Primary Care, Faculty of Health, Medicine and Life Sciences, Maastricht University, Maastricht, The Netherlands

**Keywords:** Defensive practice, Low value care, Medical professionals, Qualitative study, Theoretical domains framework

## Abstract

**Background:**

Defensive practice occurs when physicians provide services, such as tests, treatments and referrals, mainly to reduce their perceived legal or reputational risks, rather than to advance patient care. This behaviour is counter to physicians’ ethical responsibilities, yet is widely reported in surveys of doctors in various countries. There is a lack of qualitative research on the drivers of defensive practice, which is needed to inform strategies to prevent this ethically problematic behaviour.

**Methods:**

A qualitative interview study investigated the views and experiences of physicians in Australia on defensive practice and its contribution to low value care. Interviewees were recruited based on interest in medico-legal issues or experience in a health service involved in ‘Choosing Wisely’ initiatives. Semi-structured interviews averaged 60 min in length. Data were coded using the Theoretical Domains Framework, which encapsulates theories of behaviour and behaviour change.

**Results:**

All participants (n = 17) perceived defensive practice as a problem and a contributor to low value care. Behavioural drivers of defensive practice spanned seven domains in the TDF: knowledge, focused on inadequate knowledge of the law and the risks of low value care; skills, emphasising patient communication and clinical decision-making skills; professional role and identity, particularly clinicians’ perception of patient expectations and concern for their professional reputation; beliefs about consequences, especially perceptions of the beneficial and harmful consequences of defensive practice; environmental context and resources, including processes for handling patient complaints; social influences, focused on group norms that encourage or discourage defensive behaviour; and emotions, especially fear of missing a diagnosis. Overall, defensive practice is motivated by physicians’ desire to avoid criticism or scrutiny from a range of sources, and censure from their professional peers can be a more potent driver than perceived legal consequences.

**Conclusions:**

The findings call for strengthening knowledge and skills, for example, to improve clinicians’ understanding of the law and their awareness of the risks of low value care and using effective communication strategies with patients. Importantly, supportive cultures of practice and organisational environments are needed to create conditions in which clinicians feel confident in avoiding defensive practice and other forms of low value care.

**Supplementary Information:**

The online version contains supplementary material available at 10.1186/s12910-022-00755-2.

## Background

Defensive practice refers to behaviour by clinicians that mainly aims to reduce their perceived legal or reputational risks, rather than to advance patient care [1]. Defensive practice is of two general types: avoidance-type behaviour, where clinicians avoid practice areas, patients or procedures considered to involve higher medico-legal risks; and hedging-type behaviour, where clinicians provide tests, procedures, referrals and other interventions ‘just in case’ they may reduce legal or reputational risks.^1^ Hedging practices—the focus of this article—constitute low value care due to the lack of clinical justification and the potential for harms or costs to outweigh the chance of patient benefit [2]. A developing body of literature reveals the negative consequences of such practices, including screening in low-risk populations [3], hospital admissions for low value procedures [4] and unnecessary specialist consultations [5]. Defensive practice is contrary to clinicians’ ethical responsibilities: it deviates from sound practice; exposes patients to the physical, emotional and financial burdens of low value care; undermines trust in the patient-clinician relationship; and contributes to a misallocation of healthcare resources [1, 6–8].

Despite these ethical problems, hedging-type behaviour is widely reported by physicians and other health professionals across many countries with varying healthcare and legal systems [9, 10]. However, empirical research on defensive practice is dominated by quantitative surveys. More research is called for, especially qualitative investigation, to better understand the drivers of defensive practice and to inform interventions to prevent this type of low value care. To date, only a few published studies have used qualitative methods to investigate the phenomenon of defensive practice among physicians. A 2017 study in Denmark involved focus groups with 28 general practitioners who described defensive practice as “counter to the GP’s professionalism and common sense”, yet is persistently motivated by a range of pressures that doctors experience in their work [11]. A 2011 interview study with 19 physicians and 4 nurse administrators involved in preoperative decision-making at an American city hospital identified medico-legal worries as one driver of unnecessary testing [12]. In New Zealand, interviews with 12 hospital-based specialists [13] and 10 general practitioners [14] revealed the negative emotional and defensive impacts of being the subject of a disciplinary complaint. A British study involved interviews with around 20 midwives and obstetricians who had completed a survey on defensive practice [15]. This research explored connections between the fear of litigation and practices such as increased record-keeping and the use of obstetric interventions during labour. Survey studies have involved qualitative analysis of open text responses, including exploring doctors’ views on the most stressful aspects of being the subject of a complaint [16] and manifestations of defensive practice in a low-litigation risk context [13].

The present study, conducted in Australia, aimed to investigate physicians’ views and experiences in relation to hedging-type defensive practice. In particular, it explored the psychosocial drivers of such behaviour, its contribution to low value care, and strategies to ameliorate the factors that drive this ethically problematic practice.

## Method

Our methodological description is guided by the Consolidated Criteria for Reporting Qualitative Studies (COREQ) [17].

### Study design

This was a qualitative descriptive study, a design suited to understanding “a phenomenon, a process, or the perspectives and worldviews of the people involved … where the experience is described from the viewpoint of the participants” [18]. In healthcare research, qualitative descriptive studies are valuable for using knowledge gained from participants to inform interventions [19]. This research was ethically approved by the University of Technology Sydney Human Research Ethics Committee (HREC Approval No. ETH18-2985).

### Participants

Two groups of physicians were chosen to obtain insights from informants with requisite understanding and experience relevant to the topics of defensive practice and low value care. The first group consisted of physicians with interests or expertise in medico-legal matters affiliated with a professional body (e.g., Australasian College for Emergency Medicine) that participates in Choosing Wisely Australia,^2^ or with medical defence organisations, which provide medico-legal support and professional indemnity insurance for doctors. The second group consisted of clinicians working at Choosing Wisely Australia champion health services. These are hospitals and other health service organisations involved in initiatives aimed at reducing low value care. Choosing Wisely Australia is a national organization—part of the international Choosing Wisely movement—that promotes safety and quality in healthcare by reducing unnecessary tests and treatments.^3^ Study invitation notices were distributed by organisations (e.g., by e-newsletter) and physicians interested in being interviewed contacted the research team. They were then provided with a detailed information sheet about the project. The broader research study on defensive practice and low value care also included interviews with healthcare consumer representatives; those data are reported separately [6].

### Data collection

Semi-structured interviews were conducted with questions organised into three main topic areas. First, participants were asked about their general views on defensive practice, including the extent to which they perceive defensive practice as a problem in Australian healthcare, its contribution to low value care, and factors they perceive as driving defensive behaviour. Second, specific factors that influence clinician behaviour were explored in more detail. These factors were organised according to the Theoretical Domains Framework, which encapsulates theories of behaviour and behaviour change to provide “a theoretical lens through which to view the cognitive, affective, social and environmental influences on behaviour” [20]. The TDF consists of 14 domains and to prepare for interviews of around one hour in length, question prompts were organised under the seven TDF domains that best reflected current evidence, based on our systematic review of literature on physicians' views and experiences of defensive practice [9]. These domains were: knowledge, such as knowledge of the law; skills, especially communication skills; emotions, including tolerance of uncertainty; professional role and identity, particularly clinicians’ perception of patient expectations and concern for their professional reputation; social influences, such as group norms in relation to defensive behaviour; organisational context, such as processes for handling complaints; and beliefs about consequences, especially perceptions of beneficial and harmful consequences of defensive practice.^4^ This part of the interview was designed to enable discussion of a range of potential drivers, including elaboration of factors interviewees mentioned in their opening responses. Third, based on identified drivers of defensive behaviour, interviewees were asked about strategies to ameliorate these factors. Our use of the TDF followed current guidance on avoiding a rigid and technical application of the domains in qualitative research [21]. Demographic details were collected on field of practice, years of professional experience, age, state/territory of work, and gender.

Interviews were conducted by the lead author (NR) in April and May 2019 for the first group of physicians with medico-legal interests, and between February and June 2020 for the second group of physicians with Choosing Wisely champion health services. Consent to participate was verbally confirmed at the start of the interview. The interviews averaged around 60 min in length, were audio-recorded with participants’ permission and transcribed verbatim by a professional transcription service. One participant chose to be interviewed in person at a clinical site and others were interviewed by telephone or web conference.

### Data analysis

A multi-stage analytical process was undertaken. First, two authors (NR and BJ) independently read the transcripts in full. Second, they re-read each transcript for coding purposes. Responses to the opening interview questions that elicited general views on defensive practice were summarised in narrative format. The TDF domains were used to code the specific influences on clinician behaviour and the strategies interviewees suggested to ameliorate those drivers. One analyst used NVivo and the other used word processing software. While we focused on the key domains as listed above, we remained open to narrowing or expanding the domains and also recognised that there is some overlap across the TDF domains (for example, beliefs about consequences are influenced by emotions and social influences). Where data potentially fit more than one domain, the authors discussed and agreed on which domain best represented the meaning conveyed in interviewees’ statements. The extracted text was then compared and discussed for a preliminary synthesis of key findings. Next, the lead author (NR) re-read the coded data to consolidate the findings into table format to depict seven dominant domains with illustrative quotations from the transcripts.

All authors participated in a final synthesis of the findings in relation to the theoretical domains and to narrow the selection of quotations according to the following criteria: representative of strong patterns in the data; discrepant quotations used purposefully to illustrate a different perspective; and distribution of quotations across participants [22]. Quotations were edited to tidy oral speech but with attention to retaining authenticity. Illustrative quotations appear in the main text and an Additional file 1: Table S1 provides additional examples.

## Results

A total of 17 medical professionals were interviewed, eight with medico-legal interests (identified as ‘ML’) and nine from Choosing Wisely Australia champion health services (identified as ‘CW’). Interviewees reported experience in a range of clinical fields, including emergency medicine, intensive care, obstetrics, pediatrics, oncology, psychiatry, pathology, infectious diseases, palliative medicine, dermatology and general medicine. Most had practice experience of around 20 years, and one had less than five years of experience. Thirteen interviewees were male and four were female. Interviewees were from the four most populous Australian states of New South Wales, Victoria, Queensland and Western Australia. Three interviewees also reported practice experience in New Zealand. This was an experienced group of informants and, given the specific focus of the study on a medico-legal topic, the in-depth interviews, and the use of established theory, adequate informational power for our study aim was attained with this sample [23].

A narrative summary of interviewees’ general views on defensive practice is provided next, followed by the findings on specific drivers of defensive practice and proposed ameliorative strategies. The data on drivers and strategies are grouped together according to the relevant TDF domains. Table 1 summarises the results and the Additional file 1: Table S1 provides additional interview quotations.

**Table 1:** Summary of results

General views on defensive practice		
Defensive practice is: of moderate to significant concern in Australian healthcare underpinned by anxieties about “missing something” and “getting into trouble” influenced more by the fear of peer criticism than the threat of formal legal sanctions just one of multiple drivers of low value care (LVC)		

Domain	Factors	Strategies
*Specific factors that influence defensive practice and ameliorative strategies*		
Knowledge	Poor knowledge of the law Insufficient understanding of harms of LVC at patient and system levels	Education on core legal standards and requirements (e.g. law requires reasonable care, not perfect care) Education to improve knowledge of LVC and recognition of downstream harms
Skills	Inadequate skills in clinical reasoning and patient communication Time constraints and patient characteristics can hinder application of skills	Training and mentorship, especially for junior doctors, in clinical reasoning and communication Recognise value of investing time to avoid LVC Support patients to develop health literacy skills
Professional role and identity	Professional identity as a “doer” leads to pro-intervention bias LVC perceived as reassuring anxious patients	Reinforce patient safety as core to physicians’ role and identity Challenge habitual practices that are of low value
Beliefs about consequences	Belief that tests and procedures will protect against negative outcomes Risks of under-investigation seen as higher than risks of over-investigation	Increase awareness of harms of over-investigation, including broader recognition of what ‘harm’ can mean for patients (e.g. physical and emotional toll, time burden, financial costs)
Environmental context and resources	Inadequate organisational support Poor processes for managing complaints and investigations	Foster collegiality and openness Ensure timely and supportive processes that address environmental context
Social influences	Desire to avoid peer criticism Conformity to culture of defensive practice and over-investigation	Challenge norms of ‘just in case’ interventions Avoid ‘blame-and-shame’ culture of practice
Emotions	Fear of ‘missing something’ Takes bravery to resist LVC	Strengthen tolerance of uncertainty Leadership and support from senior clinicians

### General views on defensive practice

All interviewees perceived defensive practice as a concern, ranging from “moderate” (CW8) to “very concerned” (ML1), or a “big” (CW5) or “significant” concern (CW3). Participants noted low value pathology tests, scans, referrals and hospital admissions as examples of defensive practice. Fear of missing a diagnosis was a common concern and doing ‘just in case’ investigations was seen as protective:

“If you get investigated for something that didn’t go well and you did all the tests that you could possibly have ever imagined doing in the entire world it’s really, really easy to say it wasn’t my fault because I did all the tests.” (CW6)

Doctors can be “very defensive with what they do in terms of the tests that they … do, because we think that's a way of saying, well I've done everything I possibly can, I couldn't have done more.” (CWC 9)

Participants also described how defensive practice is a response to general anxiety about “getting into trouble” (ML5), which encompassed criticism by other doctors, patient complaints, internal investigations, external reviews, such as by a disciplinary body or coronial inquest, and negligence litigation. Fear of being criticised by colleagues or superiors was cited as a more powerful influence than the risk of formal legal claims, but less openly discussed: “I think it’s easier to talk about our fear of lawyers than to talk about our fear of looking bad in front of each other.” (CW6).

Doctors’ level of concern about medico-legal risks was considered disproportionate to the actual chance of being involved in a legal claim: “there’s a lot of talk about the legal risk but I actually think the legal risk is really overstated … the whole system has created a fear that is out of proportion to the likelihood of it [a legal claim] happening or in fact the outcome when it does happen.” (CW6).

At the same time, legal consequences were seen as playing a necessary role to deter substandard practice and provide redress for patients: “I defend the right of the patient to sue [doctors] because … we need to be accountable for what we do.” (ML5) “For the ‘normal doctor’ who practices good medicine, they have nothing to fear. Vexatious complaints will be weeded out. For doctors who are rubbish, they should be worried about complaints. Their practice should be restricted or reviewed.” (ML2) However, a balance must be struck: “it's hard to balance too many complaints with not the right amount.” (ML4).

Certain types of practice settings and patients were perceived as carrying higher medico-legal risks. For instance, doctors may be inclined to practice defensively in “some specialty areas where they're meeting people for the first time with potentially high-risk [conditions]” (CW8) or when seeing patients with complex comorbidities where “it’s hard to know who’s really in charge” (CW8) of managing an overall plan of care. Patients with non-specific symptoms such as headaches, or generic abdominal or chest pain can also provoke ‘just in case’ tests and procedures.

Patients perceived as having unbendable expectations can influence a defensive mindset:

Feeling that “I need to be careful with this particular patient or family ... I'm going to do more tests to show that I'm being absolutely as thorough as I could … then segues into practicing much more defensively and doing tests that are perhaps not so appropriate.” (ML1)

Most participants, however, found that a small minority of patients resist evidence-based discussions about whether tests, procedures or treatments are warranted and most are open to such conversations:

“I think we do have certain categories of patient that expect all kinds of tests to be done. … Some patients in that category are just - you can't change their mind at all. But most of those people that … take this level of interest in their own medical condition are also the people that are quite happy to have a chat and have a good discussion about things and try to understand things.” (CW1)

Where a doctor has an existing relationship with a patient, and/or the patient is seen as less anxious, doctors felt more comfortable avoiding low value care, for example, recommending a ‘watch and wait’ approach before doing further investigations.

Fear of legal risk was generally perceived as just one of multiple drivers of low value care; “it’s seldom a dominant or the only factor.” (CW7) Other drivers of low value care included habitual patterns of practice (“A lot of it is driven by fearful medicine, a lot of it is driven by lazy medicine.” (CW9)), financial incentives (“Unless there's some sort of financial restriction on your ability to do the certain test, I don't think you're ever going to reduce the use of them.” (CW5)), and availability of technology.

### Specific factors that may influence defensive practice and ameliorative strategies

#### Knowledge

Interviewees commented that many doctors have poor knowledge about the law, which can be perceived as capricious and draconian: “clinicians are very wary of the law” (CW9) and they “don't really understand the law. They also feel that if a complaint is made then it [the outcome] is a lottery.” (CW8) Improving knowledge of the law was seen as beneficial to increase clinicians’ confidence in their decision-making, and to provide reassurance that the law operates on a standard of reasonableness, not perfection, and does not penalise every poor outcome:

It would “be enormously reassuring to many doctors [to understand that] the law is not asking for the unreasonable. The law is founded on what is reasonable.” (ML1)

“Everyone can miss things; everyone will miss things. It’s impossible not to miss things. … Medicine is not 100% infallible. … To be honest, sometimes you can make a genuine mistake which everyone else in your setting would have made as well. … You're not held to the account of the best person on earth … you're held to the standard of a reasonable peer.” (ML4)

“The more [doctors] understand the boundaries of the law and what they need to do, the more confident they become in their decision-making.” (ML2)

In regard to knowledge of strategies to reduce legal risks, interviewees commonly cited effective patient communication, staying up to date on current evidence and practice guidelines, seeking peer advice in uncertain situations, and good documentation: “The best protection is communicating and being open with your patients.” (CW7); “We don't always get it right, but if we document clearly our thinking and justifications for what we've done, then there's no more we can do and that should be a protection for us against any serious litigation.” (CW9) Given the multiple drivers of defensive practice, education about the law is not sufficient to fix the problem: it is “naïve [to think] if only doctors understood the law they wouldn't work that way.” (ML8).

Interviewees stressed the need for more knowledge and awareness of the potential harms of low value care. They observed that doctors tend to focus on the potential benefits of tests and treatments: they “downgrade the severity of perceived risk and upgrade the upsides.” (CW8) Participants discussed a range of harms arising from over-investigation and over-treatment, encompassing physical harms, emotional burdens and financial costs to individual patients. Systemic harms due to the misallocation and poor stewardship of healthcare resources were also recognised. “Everything we do, there is a cost, whether it's procedures, an investigation, there is a cost, to the person, to the system.” (CW9) Dealing with incidental findings was cited as a particular problem where cascades of investigations and treatments expose patients to avoidable risks: “The more you go looking the more you’ll find,” (ML3) and this is “a massive, massive problem … [doctors can order] lots of scans and not think about the incidentalomas they're going to find and then have to follow up and you're putting the person into the health system forever when they don't need to be.” (CW5).

Yet, the downstream harms tend to be minimised or are invisible:

“[Doctors’] awareness of downstream complications is - unless it's a catastrophic thing, is almost unseen. … They [patients] go and have another test, they feel worried for a while, while they wait for the tests. They pay extra money; they get a bit of radiation. Most clinicians think, well that's the end of the harm, and it's a small number of people who will end up with the [complication that] lands them in hospital, or the unnecessary biopsy that causes them to bleed and end up in ICU … I'm not sure that the feedback of those downstream harms comes back to the initial instigator as well as it could.” (CW8)

Improving knowledge and awareness of the harms—and attendant legal risks—of unnecessary interventions was identified as important to shift doctors’ behaviour: “…. If you're doing something which isn’t clinically indicated and leads to some sort of patient injury, any claim arising out of that injury is going to be completely indefensible.” (ML8).

#### Skills

Strong skills in clinical reasoning and communication were cited as essential for avoiding defensive practice and low value care, especially for patients with non-specific symptoms. “There can be an assumption that all doctors will do multiple blood tests, multiple investigations… [but] the main [question is:] Is it going to change our treatment?” (ML1) Lack of experience and confidence can limit doctors in the exercise of these skills: “the more junior the person is, the less confident they are in their own clinical acumen so therefore they will use tests as a kind of fishing expedition.” (CW4).

When asked whether doctors are equipped to explain concepts such as over-diagnosis and overtreatment with patients, one interviewee responded: “I think you’d find very few doctors that would say they’re not skilled enough to communicate. I think that time is the issue. Some may have an attitude that information between a doctor and a patient is—goes in one direction only … rather than to share and discuss.” (CW7) On the issue of time, interviewees had divided views. Some felt that doctors have time to discuss why certain interventions are not indicated, but it is simpler to order tests, procedures and referrals. Others felt that time pressures were difficult to manage, especially with a heavy patient workload: “If we had more time we could not do the investigations.” (ML7); “Often, to be honest, addressing it [low value care] takes a lot of time which we don't have. … If someone appears anxious sometimes it's actually faster to do a scan … because you've got to see the next person.” (CW5) Another interviewee commented that investing time in patient education and counselling can save the time that would be spent on unnecessary investigations and follow-up:

“I think that's a myth [that conversations take too much time]. …If I sit down with a patient for 20 minutes and then don't decide to do certain investigations, an investigation like a CT scan often takes - at least in our hospital - close to three hours between requesting it, having a scan done and being recorded. That's three hours further. I think there is time [to have the conversations] - the time just needs to be spent more efficiently.” (CW1)

Patient characteristics were also cited as influencing how doctors exercise these skills. Doctors described how some patients were very open to conversations about current evidence and their options, while others are more passive or deferential to a doctor’s recommendation. Interviewees acknowledged that medical consultations can be stressful, patient recall of oral information is low, and it is important to use multiple communication strategies, including written summaries, high quality information sheets or website resources. Patients need support to develop their skills in understanding and evaluating information and being active in decision-making: “there's a lot of pros for patient empowerment [but people need] abilities to be an empowered patient.” (ML4).

Some patients may have specific concerns or expectations based on information from ‘Dr Google’. Online misinformation and social media stories can worsen patient anxiety and clinicians need skills in re-orienting patients back to their own situation. One interviewee described their approach to patients who have “Googled a lot and they've seen many, many stories and they're very troubled by what they see … So what I tend to do—which is a very simple thing—I say, ‘I wouldn't look too much more at those. Each person is different, and I'm going to follow you. This is your story, it's not another person's story.’” (ML1).

#### Professional role and identity

Interviewees described the professional role perceptions that are implicated in the provision of low value care. A professional identity as a ‘doer’ who can solve patients’ concerns may promote a pro-intervention bias: “doctors are doers … in general it’s much easier to do something rather than just not do anything” (ML8); “… doctors do a test to show they are doing something. They are aware of patient expectations and concerned about not meeting them.” (CW4).

Acceding to low value tests and procedures to reassure patients can create professional role tensions and efforts to justify the behaviour: “It's not really defensive medicine because we have to do the scan to alleviate their anxiety. But it still is defensive medicine because we shouldn’t be doing it, but in a way it is therapeutic to the patient.” (ML7) In addition, a professional role tendency toward perfectionism can be counter-productive:

“There's a degree of perfectionism in medicine … living with uncertainty can be difficult, particularly if you're a perfectionist, where you think, I want to uncover everything - I want to turn over every stone. That then leads you into potentially into doing more and more investigations, where your perfectionism is starting to crowd out common sense.” (ML1)

Doctors’ biases toward tests and procedures can influence patient beliefs about optimal patient care: patients “say I'm so lucky I have such a good doctor, she's so thorough. She does every single investigation and if she's worried in any way then she sends me for a CT that does head to toe. I have the best doctor in the world.” (ML5).

Several interviewees reflected on a shift in medicine where the patient is now a “customer”, and this can change the professional role: “If they're a customer you give them what they want. But, of course, giving them what they want isn't necessarily the correct thing to do from a professional point of view.” (ML8) Another interviewee observed: “Doctors have contributed to that problem because we have become, as a profession, so mercenary and so transactional, and we've lost that relational theme.” (ML5).

To avoid defensive practice and low value care, interviewees emphasised that patient safety must be at the core of a doctor’s role and identity, challenging habitual practices that are of low value, recognising the harms of low value interventions and supporting patients to be active participants in their care:

“How you define being a good doctor is giving the patients information … to support medical care which is in their interest. … how I would define being a good clinician is having that conversation and letting them become a participant in their own health care rather than a more paternalistic sort of approach ... [of] giving them instruction rather than information.” (CW7)

#### Beliefs about consequences

Beliefs about consequences related to the view that ordering tests and procedures for a patient offers protection from negative consequences: “if you over test you don’t get in trouble; but if you under test you do.” (CW5) Beliefs about consequences were described as being strongly influenced by prior negative experiences, such as missed diagnoses that result in poor patient outcomes, criticism from superiors and peers, and legal repercussions:

“If … you didn’t do all the tests because they weren’t indicated and you were really unlucky that this was the one in a million [where you missed something] - it’s very, very hard to defend that. I don’t mean medico-legal defend it, although that might become an issue. It’s more that it’s hard to defend it to the [internal] team investigating and to your colleagues and your peers who will be talking about it behind your back.” (CW6)

Worst cases scenarios also attract more attention than good outcomes, influencing a tendency to catastrophise and fear the worst.: “It only takes one patient where you've ignored a vague symptom … and then … later … they've got widespread metastatic disease. We've all been burnt—and it’s those cases which we’ve all had that make us wary in the future.” (ML7).

Failing to take account of the harms of low value care also involves skewed beliefs about consequences. Extensive investigation of every non-specific symptom may identify some unsuspected diagnoses but “end up actually causing a huge amount of harm … [for] the vast majority of patients.” (CW6) Yet, as noted earlier, these harms were seen as under-acknowledged.

#### Environmental context and resources

Interviewees described the importance of supportive environments to reduce defensive worries. Doctors need to be able to seek colleagues’ opinions, especially in difficult patient cases, and they need opportunities to de-brief when things go wrong. Organisations also need to be consistent in messages and actions; if a hospital promotes initiatives to reduce low value care, they also need to support doctors who face criticism or complaints because they made a reasonable decision to not order a test or procedure:

If “the organisation on the one hand pushes Choosing Wisely, but then on the other hand you're just not sure if they will back you up if something happened and you hadn't done a test. I think it's very hard to defend why you didn't do a test. It's much easier to defend why you did.” (CW5)

“There is a definite concern that even if we believe we've done our very, very best, that they will still come for us and that the hospital may not support us.” (CW9)

Negative experiences can drive future defensive behaviour. Clinicians “want to avoid complaints. They're a nightmare. They’re stressful … A lot of doctors will do anything to avoid them.” (ML7) For example, if a clinician’s decision not to order a test is questioned:

“you would now be doing those tests on every single patient … because you’d be so terrified of going through that [investigation] again. How we run those investigations, whether they're clinical or legal, is so important.” (CW6)

Doctors who have confidence in such processes were considered less likely to practice defensively, especially if vexatious complaints are addressed quickly to avoid negative impacts on doctors’ professional reputations. When dealing with complaints and investigations, an important goal in terms of environmental context is “trying to understand how good staff with good skills ended up in a situation that wasn’t good for the patient.” (CW6).

#### Social influences

Interviewees commented on the influence of colleagues, noting that defensive practice can be driven more strongly by a desire to avoid the scorn of other clinicians than to avoid legal claims by patients. Medicine was described as a competitive profession and doctors do not want to get things wrong in the eyes of their peers:

“What's quite common in our setting is to be crucified by your colleagues.” (CW6)

Doctors are “more worried about their loss of prestige amongst their colleagues. So they may order excessive tests because they don’t want to be seen as missing something. But that’s not … in the patient’s interest, it’s about their own prestige within the group. … What is defined as a good … physician by many is, have you thought of every possible low probability case.” (CW7)

“It's a fear of criticism. It's not a fear of being sued. … I remember when I [had a very poor patient outcome]. My anxiety totally related to … how my peers would view me. So how the court would judge me … that's judged by a completely different standard, and I respect that standard.” (ML5).

Cultures of practice within healthcare organisations were described as encouraging conformity to group norms. The norm can involve risk aversion and over-testing or it can involve active practices to mitigate the drivers of defensive practice and low value care. Describing both ends of this spectrum, one interviewee commented that defensive norms can override clinical judgment: “the clinical indication might be to do X. The defensive, lower risk, self-preservation practice might be to do Y, and we often do Y.” (CW3) But in a supportive culture, “there is an acceptance, culturally, in the peer group that risk is inevitable and that misadventure is inevitable. … So, there’s probably not a blame-and-shame culture.” (CW3) Other interviewees expressed concern about the mentality that it is “better to do a test, just in case” and “you have to do a test to rule out the ‘unicorn’ diagnosis,” (ML3) referring to rare conditions. When physicians discuss cases involving rare diagnoses, the message often conveyed is:

“how awesome was this doctor because they found it. So I think it’s about how we present and discuss those cases as well when we do find the unicorns as well as when we miss them. Because I think when we miss them we annihilate people for missing them. But when we find them we all tend to take an awful lot of credit and I think the message [about avoiding over-investigation] gets lost along the way.” (CW6)

#### Emotions

Interviewees described a range of emotions that underlie defensive practice, especially the fear of missing a diagnosis or problem: “As a doctor you really are genuinely scared of making a mistake. You are making the decisions that affect people’s lives and you really are genuinely scared of … getting it wrong.” (CW6) Junior clinicians have the added emotional burden of the “fear of disappointing their superiors” (CW8) and “the wrath of senior clinicians.” (CW9) These anxieties can create a “culture of fear” (CW6) and emotional fortitude is required to resist doing low value tests and procedures:

“It takes bravery because often there's this feeling of uncertainty and you're just - you're very scared of missing something. In that sense it takes a certain bravery to not do a test.” (CW1)

“Doctors say to me that they need to be brave. That they are somehow stepping outside of the norms just to say, no we need to stop and question some of these things [low value tests and procedures].” (ML4)

Several interviewees described the confidence that comes with experience: “… in the early stages of our career … we tend to over investigate. As we get more senior … we're a bit more confident and rely on our clinical skills. … In my first 10 years … my level of defensive medicine was exceptionally high.” (ML7) However, being the subject of a complaint can have longstanding emotional impacts, even for experienced clinicians: “the older you get the more comfortable you get with more uncertainty I think; unless you've had complaints and then that changes you forever.” (CW5).

How mistakes or poor patient outcomes are discussed can heighten or decrease anxiety:

I think when we talk about our mistakes … it probably does make them [other clinicians] worry more that they may well do the same thing … discussing mistakes is a bit of a double-edged sword, but it's important for… us to understand that they will happen in a very, very complex environment, but we need to look at what we can learn. (CW9)

Another interviewee stressed the importance of peer support and de-briefing when things go wrong:

There's no acknowledgement of the emotion [when there has been a poor patient outcome], and that it's, okay, next, next, next - so you're moving on to the next patient. No one really sits and talks about the emotional dimension of what they've seen or done - in other words, the whole thing of debriefing, really, can be very, very important - but that does require seniors to show the leadership, to say, in our department, we're going to do this. (ML1)

## Discussion

### De-implementing defensive practice

Literature on defensive practice and the de-implementation of low value care identifies barriers, facilitators and potential intervention points at the micro level of individual clinicians and patients, the meso level of social and organisational contexts, and the macro level of legislation and public policy [9, 24]. A striking finding from the present study is that physicians described defensive practice as a means to avoid criticism or negative interactions emanating from a range of sources. Criticism by colleagues was cited as a particularly powerful driver, more potent than a perceived threat of legal consequences. An important implication is that social and organisation level strategies that tackle peer criticism factors are essential for clinicians to feel confident in discarding defensive practices.

De-implementation research has focused on factors at the level of the individual clinician with comparatively less attention to social factors that influence the provision or avoidance of low value care [24]. As we discuss, there is a role for strengthening clinicians’ knowledge and skills, but these individual characteristics can only be put to good use in supportive social and organisational environments [25]. The fear of having one’s decisions scrutinised by colleagues also offers an explanation as to why law reforms aimed at reducing malpractice liability risks have had limited impacts on defensive practices [26]. Even if such reforms offer formal protection from litigious patients, they do not shield doctors from the censorious judgement of other clinicians.

In the following discussion, we focus on several factors—legal knowledge, attention to the risks of low value care, patient relationships, and supportive environments—and their significance to de-implementing defensive practice.

#### The significance of legal knowledge

Participants in this study agreed that doctors often have poor understanding and skewed perceptions of the law. However, deficiencies in legal knowledge are not unique to doctors. A review of empirical research on knowledge of the law among professionals and lay people across various areas, including healthcare, concluded that “ignorance and misunderstanding of the law is common” [27]. Our findings build on van Rooij’s proposition that, in the face of legal ignorance, “people tend to equate their own norms with the rules of the law” [27]. Doctors may inaptly equate the standard of perfection that they impose on each other as the standard the law expects of them.

This perfectionist standard is conveyed in various ways and reinforces cognitive biases that favor defensive over-investigation and over-treatment [28]. For example, clinicians perceive it is easier to justify doing a test, rather than not doing it. Refraining from sending patients for diagnostic investigations may be seen as ‘under-testing,’ which implies behaviour that falls below an acceptable standard. Participants described the need for bravery to stand up to the threat of criticism from colleagues and to question low value tests and procedures.

Understanding these influences on doctors’ practices helps to explain why some research shows that clinicians worry that following Choosing Wisely recommendations to avoid low value care will *increase* their medico-legal risks [29–31]. From a legal perspective, following reputable, professionally developed and endorsed guidance helps clinicians refute claims of substandard practice. However, if clinicians feel they are stepping outside accepted local norms by *not* ordering tests and procedures, they then open themselves up to the risk of being ‘crucified’ or criticised by other practitioners.

Previous research posited that “legal defensiveness and knowledge of medical law are inversely related” [32]. A study involving Australian medical professionals indicated that misperceiving the law as imposing unattainable standards resulted in “hostile” attitudes about the law [33]. In other research with clinicians involved in palliative and end of life care, those with more accurate legal knowledge had more positive attitudes about the law and were least likely to be worried about legal risks [34].

Participants in the present study agreed that education to strengthen doctors’ legal knowledge would help to alleviate exaggerated worries that contribute to defensive practice. They described the reassurance of understanding that the law operates on a standard of reasonableness, not perfection. Can education improve knowledge and improve practice? This question requires further research, but recent studies suggest positive outcomes. Targeted legal education interventions, such as a 1-h training session on consent-to-treatment law, have improved clinicians’ knowledge, at least in the short term [35]. In the United States, an education program for medical residents addressed fear of legal risks as a driver of low value care and showed sustained improvements in avoiding defensive practice [36]. Education alone will not fix the drivers of defensive practice. However, improving clinicians’ understanding of key legal principles may help to supplant the informal norms that emphasise doing everything possible in the quest for certainty in diagnosis and care, to the admiration of peers who endorse this behaviour.

#### The significance of attention to the risks of low value care

Improving understanding of the law *and* of the harms of low value care were intertwined messages in our study. From a legal perspective, defensive practice can *increase* the chance of complaints or litigation because patients are exposed to the risks of avoidable harm, including cascades of tests and procedures, hospital-acquired complications and extended hospital stays. It may be difficult to gain traction with this message if the threat of peer criticism looms larger in practitioners’ minds than potential harms to patients that may or may not materialise. However, our study participants emphasised clinicians’ professional role and ethical duties in ensuring patient safety. Evidence of harms, and patients’ accounts of the impacts of these harms, can help to counter the cognitive biases that lead to overestimating the benefits of tests and procedures and underestimating the risks [37].

#### The significance of patient relationships

Prior research on defensive practice depicted an adversarial framing of the doctor-patient relationship; when asked in surveys, many clinicians agreed that they see patients as potential complainants [9]. Clinicians may also over-perceive patients as ‘inconvincible’ about avoiding unnecessary care. For example, some clinicians in a US study found it simpler and less emotionally draining to prescribe an antibiotic instead of educating a patient why an antibiotic is not indicated in their situation [38].

Participants in the present study acknowledged role perceptions that lead to low value care, such as perceiving a ‘good’ doctor as one who does extensive investigations. Yet, acceding to patient requests for unnecessary care was recognised as counter to professional responsibilities. An encouraging finding is that participants considered most patients are open to discussions that avoid unnecessary tests and treatments, and generally found a small minority of patients to be very anxious or demanding. This finding aligns with prior research suggesting that the ‘demanding’ patient is more myth than reality [39].

Participants described the importance of fostering a therapeutic relationship where patients are supported to participate effectively in their own care. Adopting simple, evidence-based practices that strengthen the clinician’s presence and connection with patients can improve both communication and satisfaction with the encounter [40]. Conversations based on “high-quality information on the clinical consequences of unnecessary … cascades can help reframe the reasons for reducing low-value care as evidence based and patient centered” [41] and with appropriate information about options, risks and benefits, “patients are unlikely to consent to low value care”[28].

#### The significance of supportive environments

Interventions targeted at clinicians and the patient interaction must be buttressed by supportive environments. The culture of medicine may be difficult to change [42], but our experienced participants identified key components of cultural change: organisational leadership; mentoring; promoting strong clinical reasoning to distinguish low and high value care; aligning practice changes with a patient safety imperative; and providing opportunities to de-brief and seek peer support when things go wrong. A recent review affirms the importance of medical leadership in de-implementation, particularly in promoting positive attitudes, good communication and team-based collaboration to change practices [24]. Different parts of an organisation must have complementary approaches; for example, training just one clinical group in a hospital on reducing low value interventions is not enough [43].

Organisations must also support clinicians who reasonably decline to provide low value care. As a study on unnecessary antibiotic prescribing points out: “clinicians should feel confident that practice administrators will support them for prioritizing judicious antibiotic use by not punishing them for negative patient reviews that mention antibiotics.” [38] It is also essential to value the time taken with patients to support informed decisions and avoid unnecessary tests and treatments. Time pressures—real and perceived—that hinder effective communication and decision-making must be remedied. Analysts contend that the “health care system must place a much higher value on and invest in innovations that create time and realize the possibility of time for patient care.” [44]

Giving up practices can cause cognitive and emotional stress for clinicians [45] and they are more likely to avoid low value care if they can replace it with higher value substitutes [37]. In relation to defensive practice, a higher value alternative is discussion with patients that builds therapeutic relationships and enables appropriately informed decision-making. Improved communication and consent processes, in turn, offer protection against medico-legal risks, as indicated by data showing that patient complaints commonly cite poor communication [46].

When complaints and legal claims are made, our findings reinforce the need for timely and fair processes and peer supports. Previous research demonstrates the detrimental impacts of complaints and litigation on health professionals’ mental health and practice behaviours, include provoking future defensive practice [16, 47, 48]. A ‘just culture’ describes an environmental context that reduces clinicians’ fears about being blamed and shamed by colleagues, and instead fosters collective learning, reflection and practices that promote safe and high value care [49]. A ‘just culture’ also mitigates the intolerance of uncertainty that can drive defensive practice: “talking openly about uncertainty in the clinical environment helps normalize the experience of uncertainty, especially for those colleagues with less experience” [50].

Our participants observed that junior clinicians are more vulnerable to the pressures that drive defensive behaviour. Defensive practices are more commonly reported among less experienced physicians [9] and a “hidden curriculum” [51] can reinforce defensive provision of low value care. For example, an analysis of training materials on the management of upper respiratory tract infections revealed messages that implicitly encouraged defensive practices in relation to diagnostic investigations and antibiotic use [52]. Strong mentorship in a just culture can ensure the next generation of clinicians are well supported to avoid defensiveness and deliver safe and quality care.

### Strengths and limitations

The participants for this study were recruited based on their expertise in medico-legal matters, thereby bringing a ‘legal risk lens’ to the interview topics, and experience in settings involved in initiatives to reduce low value care, thereby bringing a ‘practice change’ lens to the topics. These key informant perspectives were a strength of the recruitment strategy. The experienced clinicians who participated in this study represent leaders for change. Their insights show the shifts that can and need to occur to reduce the drivers of defensive practice and create conditions for safe and high value care.

A majority of participants were male, a limitation that is due in part to the level of seniority of those who volunteered to be interviewed. The participants provided insights from a range of clinical perspectives, however, junior clinicians and those who are less familiar with medico-legal issues and Choosing Wisely initiatives may have other views. Specific clinical fields and practice settings involve particular factors that drive defensive behaviours and will have differing barriers and enablers that affect de-implementation strategies.

The use of the Theoretical Domains Framework to guide our data collection and analysis also has strengths and limitations. The TDF provides a comprehensive synthesis of theories with domains that span the cognitive, emotional, attitudinal and socio-environmental influences on behavior. As an inclusive, rather than selective, framework, the TDF can reveal behavioural drivers that may be overlooked by atheoretical approaches; however, we were mindful of the need to avoid an overly rigid application of the framework [21]. We found that our specific data were well represented by the identified domains; our study is a classic ‘fit’ for the TDF with our focus on behavioural drivers and change strategies for health professionals. We recognise the value of inductive, ‘non-TDF’ approaches [21], as we applied in our exploratory study of healthcare consumer views on defensive practice [6].

## Conclusion

This study adds to the limited qualitative research into defensive practice among physicians, with a focus on the drivers of this behaviour and its contribution to low value care. The findings reveal the range of pressures and feared consequences that influence the defensive ordering of tests, treatments and procedures. Fear of criticism from peers and superiors figures as a more forceful influence than anxiety about formal legal liability. Clinicians must be “brave” to resist the pressures that lead to defensive behaviour. Individual-level interventions to strengthen knowledge and skills are worthwhile, but avoiding low value care should not be left to the fallibility of individual resistance. This study reinforces the importance of supportive environments that establish a just culture and promote ethical behaviour.

## Supplementary Information

**Additional file 1.** **Supplementary Table** – Quotations from Interviews with Physicians.

## Data Availability

The data generated and analysed for this study are not available due to participant confidentiality reasons. Reasonable requests for further information may be directed to the corresponding author.

## Footnotes

1 Other terms that appear in the literature are ‘passive’ or ‘negative’ defensive practice for avoidance-type behaviour and ‘assurance’ or ‘positive’ defensive practice for hedging-type behaviour. These latter terms may imply beneficial actions so we prefer the term ‘hedging’ behaviour, as proposed by Bourne et al. The impact of complaints procedures on the welfare, health and clinical practise of 7926 doctors in the UK: a cross-sectional survey. BMJ Open. 2015;5(1):e006687–e006687.

2 See full list of professional colleges, societies and associations here: https://www.choosingwisely.org.au/members-supporters/colleges-societies-associations.

3 For more information, see https://www.choosingwisely.org.au/what-is-choosing-wisely-australia and https://www.choosingwisely.org.au/what-is-choosing-wisely-australia/choosing-wisely-internationally.

4 The seven remaining domains are: beliefs about capabilities; optimism; reinforcement; intentions; goals; memory, attention and decision processes; and behavioural regulation.
