# Supplementary material for: A qualitative interview study of Australian physicians on defensive practice and low value care: “it’s easier to talk about our fear of lawyers than to talk about our fear of looking bad in front of each other”
Source: BMC Med Ethics. 2022 Mar 4;23:16. doi: 10.1186/s12910-022-00755-2 (PMC8895622; doi:10.1186/s12910-022-00755-2)
Supplement: Supplementary file 1 — Additional file 1. Supplementary Table – Quotations from Interviews with Physicians. [file 12910_2022_755_MOESM1_ESM.doc]

**Supplementary Table – Quotations from Interviews with Physicians**

Note: These quotations are organized according to relevant domains of the Theoretical Domains Framework. They are

drawn from interviews with physicians with medico-legal interests (noted as ‘ML’) and physicians from Choosing Wisely

champion health services (noted as ‘CW’).

| **Key Domains and Illustrative Quotations from Interviews** |
| --- |
| **Knowledge** |
| **Knowledge of the law and legal risks**  “Mostly, doctors would not have a knowledge of any depth about the law itself.” ML1  There is “profound ignorance amongst all the doctors I've ever met of what the law [requires].” ML6  “We are the most ignorant, probably, professional group when it comes to law. … Doctors don't understand the law … this is what leads them into trouble.” ML7  “The more junior the doctor, the more unknown the law is. The law is mysterious. They get scared and just do all the tests.” ML2  “Doctors are not taught very much law. [They perceive] the law is going to be immediately draconian, where in fact, the law is going to look carefully at what's gone on and listen very carefully to what you've said or done.” ML1  “I think people do get worried about missing things, and they don't really understand that ... the risk [of legal repercussions] is extremely, extremely low if … you talk to your patients … about the risks. … You do what other people think is accepted practice, if you follow those things, you pretty much are not going to have any problems. I think people jump to other stories, where [clinicians] missed the rare thing, and therefore, they did the wrong thing, and they're liable.” CW8  **Medical knowledge of the risks of low value care**  Doctors need to be “up-front with ourselves and with our patients and their families about the risks of admission … hospital-related infections and hospital-related adverse events. … Every hospital admission is a potential chance to be exposed to medication error.” CW3  It is important to consider “what the harm is in doing tests that aren’t indicated. What the harm is in over investigating. What the danger is to their patient. … So it’s really about opening up that conversation about why we do the tests we do. But equally why we shouldn’t do tests that we shouldn’t do and really making that harm really, really visible.” CWC 6  With “scans, I don’t think they're [doctors] thinking too hard about the ramifications. I think they are thinking, look I'm just going to be sure … this is going to reassure me. Yeah, they're not necessarily thinking about, oh my goodness, look what else I've turned up and I don't know what to do with that now, or I've given them radiation they didn't need or … they've had an allergic reaction to the contrast.” CW9  Doing unnecessary scans is “wasteful, and in fact sometimes it's harmful because you're getting information that is misleading or spurious or incorrect.” ML5 |
| **Skills** |
| **Communication and clinical reasoning skills**  “Communication is really important … sometimes you can go into … jargon and then [its] too technical for [patients] to understand … breaking it down into very simplistic words for them to understand is really important and then I find what really works is … allowing the [patient] to make a decision with you, rather than you telling them” what to do. CW1  “When complaints happen it is less about the clinical management and more about communication and lack of respect in behaviour.” ML2  “The person who is least likely to be sued is the most effective communicator.” ML5  “So people come in with abdominal pain, no specific findings, no specific concerns. No red flags as we say. Nothing that makes us think there’s something dangerous going on today. … I actually say that to the patients. Is there any evidence that this is dangerous to you right now? There’s not. … Let’s just sit tight … it may or may not ever come back or happen again.” “[Are test results] going to change whether you admit them or discharge them? ... I really encourage people not to do tests unless it’s going to change their management.” CW6 |
| **Social/Professional Role and Identity** |
| **Professional identity as a ‘doer’, influenced by perceptions of patients**  Some doctors “may do it [order tests] when there’s uncertainty and they feel that something needs to be done or a sense of helplessness so they will do something to give a sense of motion and activity.” CW7  “The GPs often send people to us to say, look I didn't think this was needed, but what you do think and then there will be times when we'll do the test just to reassure.” CW9    “People presenting with … non-specific symptoms [such as] a bit of fatigue and … some of these patients are very convinced they have a medical problem … I don't need to do [a test] … but they're not going to be happy until I do that. … There's got to be a limit to treating their anxiety with multiple tests.” CW5  “Some patients … are far more litigant than others. You know who are going to be the cool ones and … as long as you explain yourself that, look, I think it’s nothing but give me a call in a month if it doesn’t go away and then we'll do a scan. But really I don't want to scan you now because it's not the right thing to do. They're going to be fine with that. They'd prefer that because they don't want scans either. But there's others that you just know that are highly anxious, and will blame you for not acting on a vague symptom.” ML7 |
| **Beliefs about Consequences** |
| **Consequences of poor patient outcomes**  “I suspect, more than anything, it would be clinical experience that influences these doctors [who practice defensively]. I think if somebody had an experience of a patient going away and then a bad outcome … that would make them risk-averse.” CW3  “When someone presents with a really bad headache, no matter that it's not likely to be brain cancer. One in 500,000 will be and if you've actually seen someone with that presentation before who did have brain cancer that clearly affects people's ability to think rationally.” ML4  “There's nothing worse than being the doctor who sees the patient with mild back pain and then two days later, they're a paraplegic or a quadriplegic. That's that terrible regret about missing something which could cause enormous damage, if not death, to somebody else. You can't underestimate that. Because doctors are there to try and help their patients and the devastation of missing those sort of diagnoses is obviously devastating for the patient, because they're the ones who live with the consequences or die as a result of it and their families have to live with the consequences, but the doctors also live with that as well.” ML8  “If you treat that very unusual case and then say, well, everybody has got the same presentation therefore I test everyone you end up actually causing a huge amount of harm because [for] the vast majority of patients … are you looking for a horse or are you looking for a unicorn? We need to design our system to look for horses but be aware that occasionally there’s a unicorn and we need to look out for them. But we should not design our systems to look for unicorns. Because most of what we find is going to be horses.” CW6 |
| **Environmental Context and Resources** |
| **Investigation and complaint processes**  “You go through a complaint and even if there is very little that needs to be changed in the clinician’s clinical skill set or behaviours, it will cause them a certain amount of anxiety for a period of time. … Individuals need to be supported through these processes … and have appropriate follow-up.” CW4  “To be honest, having had complaints made against me, … the medical board process is so horrendous even when you haven't done anything wrong. It's just so time consuming … my personal experience is that it's taken at least six months to have a fairly simple matter sorted.” ML4  When things go wrong, we “look at both the individual factors and the system factors to try and understand what happened and to try and design our system around making the right decision the easiest decision to make and test ordering is one piece of that entire puzzle. But the more that people don’t trust the people reviewing their cases and the more that they believe that they're going to get in trouble if somebody reviews it if they didn’t do a test, the more difficult stopping people ordering test is going to be.” CW6  “Encouraging peer support [is important] … the most dangerous practitioner is the isolated practitioner, both clinically and in terms of medicolegal risk.” ML5 |
| **Social Influences** |
| **Culture of practice and reputation among colleagues**  “The dominant thing is the culture of practice. [Medicine is] a mentor sort of environment where people learn off each other’s practices. A new team member is – it’s an implicit assumption that they’ll follow the lead of their seniors … This is like a long line of tradition and it’s discouraged often to question that. I think it gives people a sense of consistency and comfort to have a … team identity.” CW7  “If the more senior clinicians are practising defensive medicine then that's passed onto the junior consultants, and then the registrars and residents, so it's a flow-on effect.” ML7  Doctors “tend to be pretty competitive. I think there’s an element in our medical culture of wanting to look better than other people and part of that comes out in the way we practice medicine. … When you talk to people about their experiences when they’ve made mistakes or had bad outcomes with patients or what they’re worried about happening, what they're worried about is somebody saying, well, you missed it and you should have done it. You should have done this test and then this wouldn’t happen. When you watch a group of doctors together they are really good at criticising everybody else. They're very, very good at saying that they wouldn’t have made the same decision and they're pretty rough on each other.” CW6  “Doctors don't like being challenged and don't like being investigated, and naturally suspicious that there's going to be a bad outcome because they're just being challenged.” ML7  “When the consultant berates them [junior doctors] because they didn’t do some random esoteric test, then what that registrar or junior learns is to do every random or esoteric test they can think of to avoid being berated by their consultant. What I think we have never really looked at was, was that test even relevant in the first place. But what it starts and what it creates is a culture of a fear of ever being accused of not doing the right tests.” CWC 6 |
| **Emotion** |
| **Fear**  “It's all driven by fear of getting it wrong and the consequences of getting it wrong.” ML5  “Some clinicians feel like the ‘sword of Damocles; is over their heads”; It is important to “dispel fear of missed diagnosis”. ML3 |
